# Supplementary material for: Ram Semen Cryopreservation for Portuguese Native Breeds: Season and Breed Effects on Semen Quality Variation
Source: Animals (Basel). 2023 Feb 7;13(4):579. doi: 10.3390/ani13040579 (PMC9951670; doi:10.3390/ani13040579)
Supplement: Supplementary file 1 [file animals-13-00579-s001.zip › animals-2199469-supplementary.pdf]

Title: Ram Title: Ram Semen Cryopreservation for Portuguese Native Breeds: Season and Breed Effects on Semen Quality Variation

Supplementary file S1

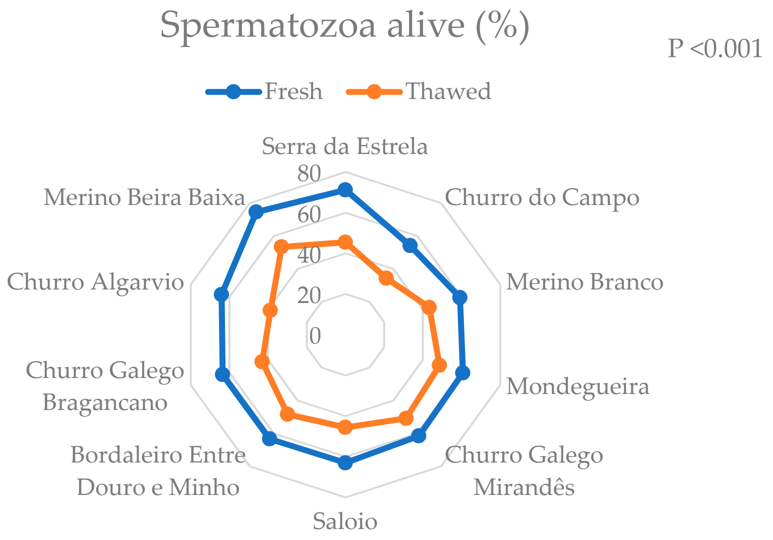

Figure S1. Breed x Thawing interaction on spermatozoa viability.

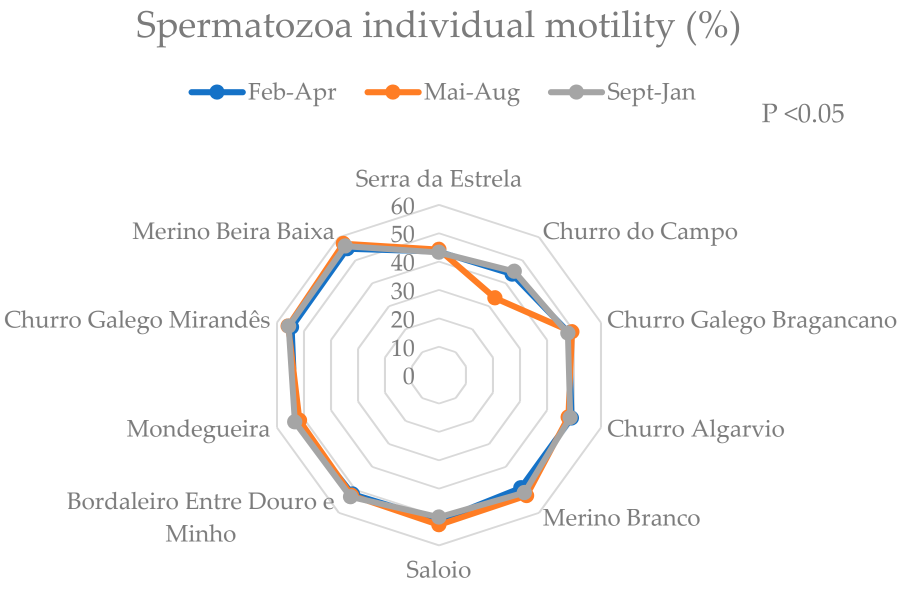

Figure S2. Breed x Season interaction on spermatozoa individual motility.

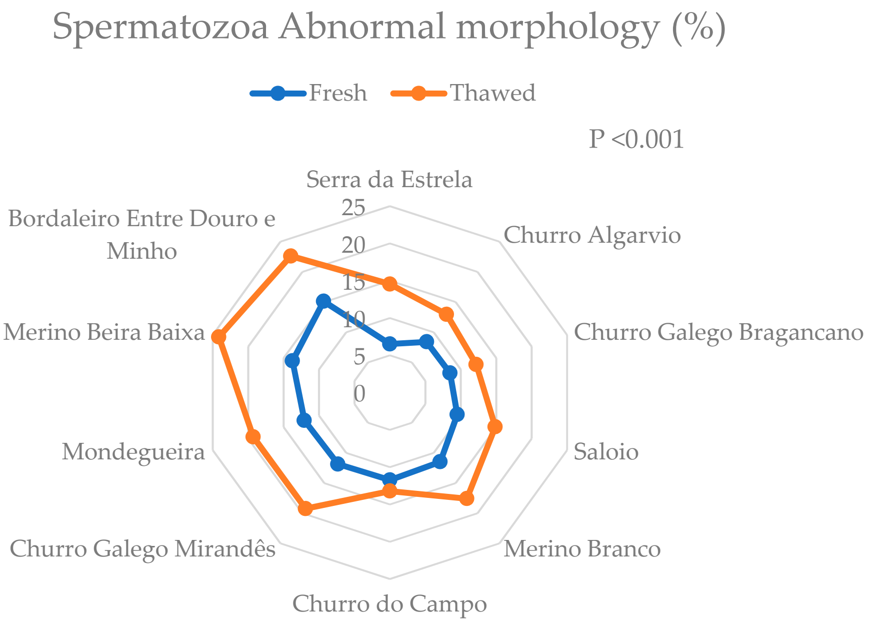

Figure S3. Breed x Thawing interaction on spermatozoa abnormal morphology.

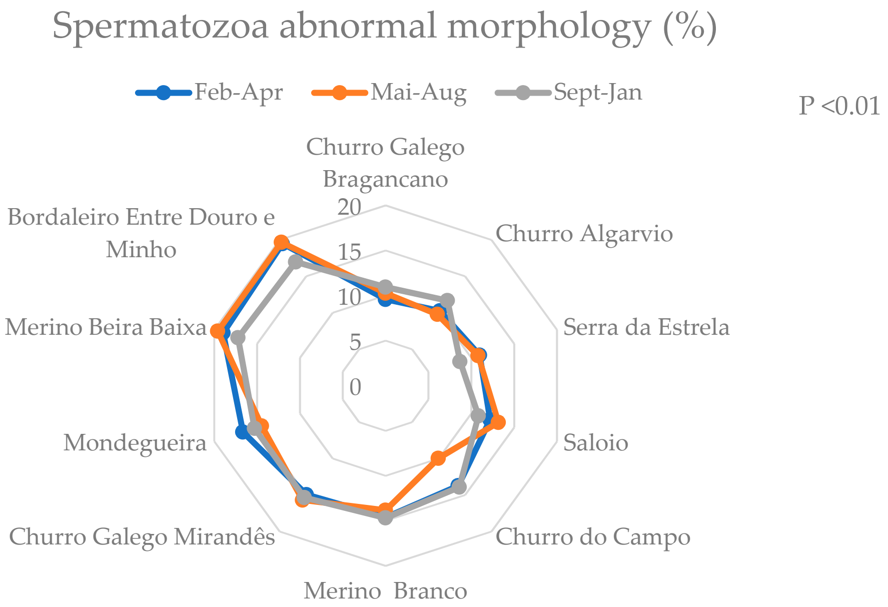

Figure S4. Breed x Season interaction on spermatozoa abnormal morphology.

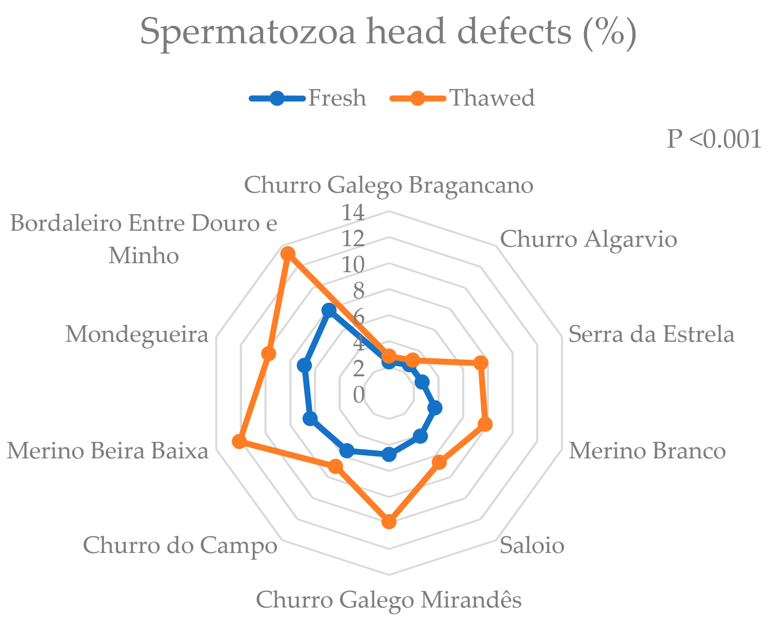

Figure S5. Breed x Thawing interaction on spermatozoa head defects.

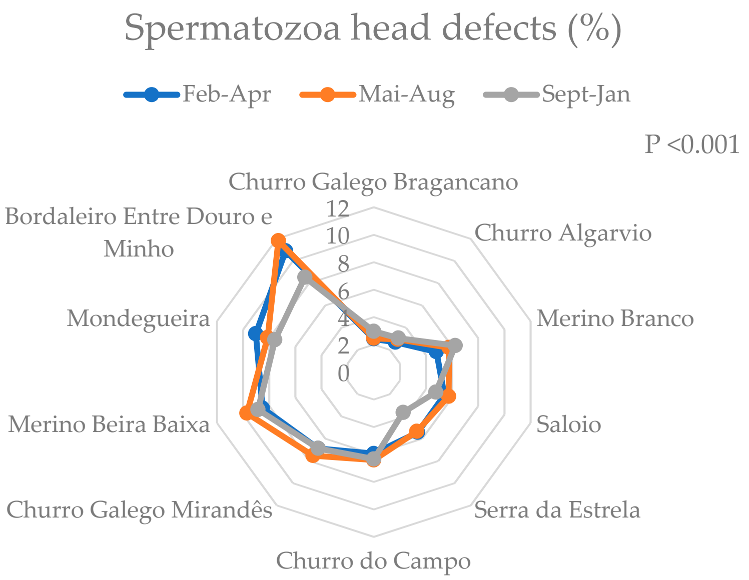

Figure S6. Breed x Season interaction on spermatozoa head defects.

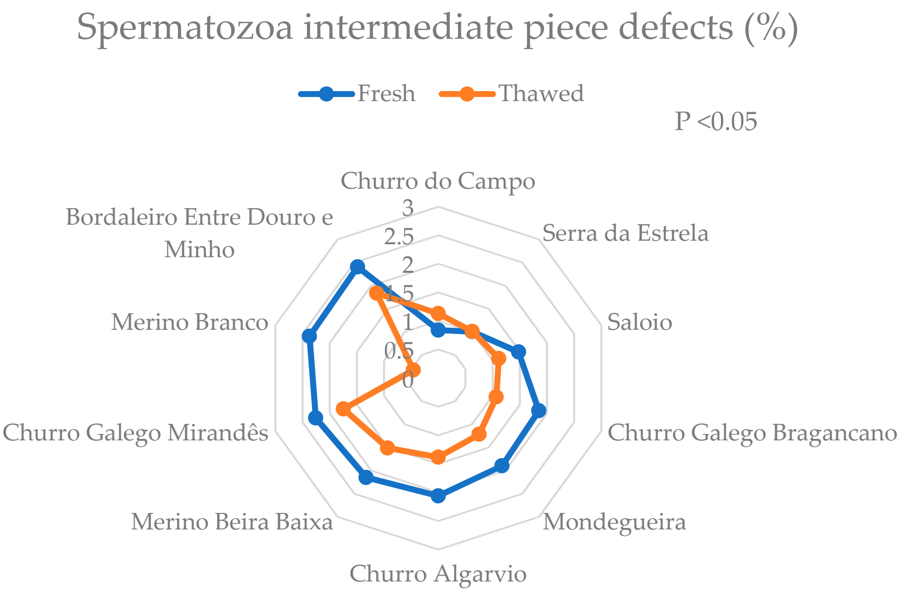

Figure S7. Breed x Thawing interaction on spermatozoa intermediate piece defects.

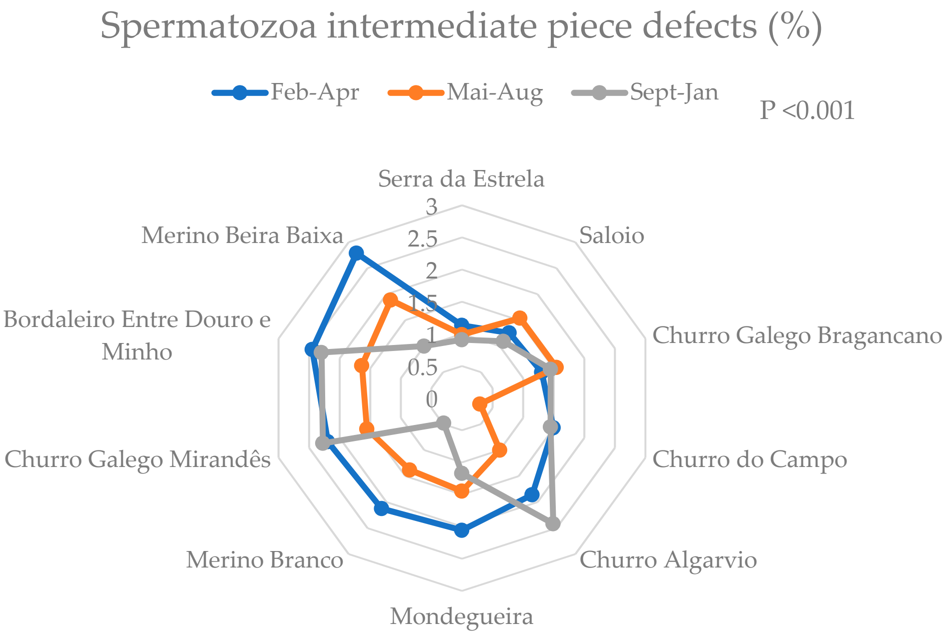

Figure S8. Breed x Season interaction on spermatozoa intermediate piece defects.

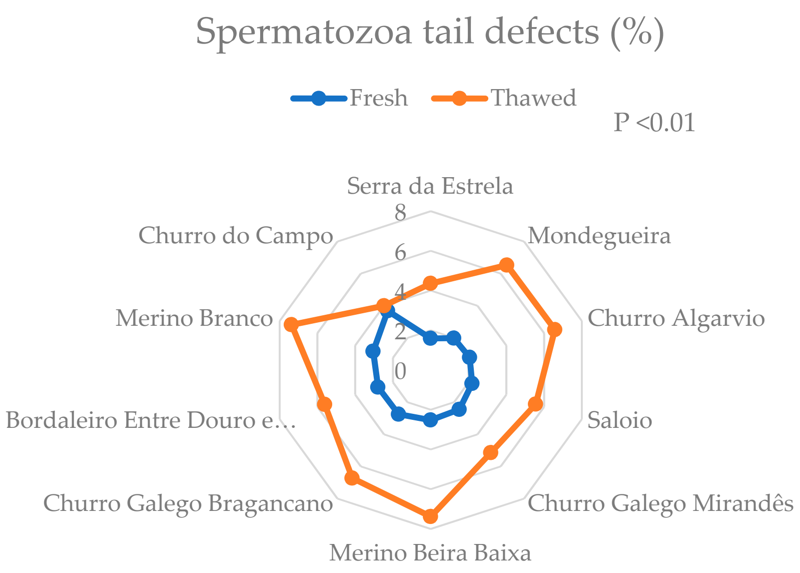

Figure S9. Breed x Thawing interaction on spermatozoa tail defects.
